# The Cytological Events and Molecular Control of Life Cycle Development of *Trypanosoma brucei* in the Mammalian Bloodstream

**DOI:** 10.3390/pathogens6030029

**Published:** 2017-06-28

**Authors:** Eleanor Silvester, Kirsty R. McWilliam, Keith R. Matthews

**Affiliations:** Institute for Immunology and Infection Research, Centre for Immunity, Infection and Evolution, School of Biological Sciences, King’s Buildings, University of Edinburgh, Charlotte Auerbach Road, Edinburgh EH9 3FL, UK; Eleanor.Silvester@ed.ac.uk (E.S.); K.R.McWilliam@sms.ed.ac.uk (K.R.McW.)

**Keywords:** *Trypanosoma brucei*, transmission, stumpy form, differentiation, life-cycle, quorum sensing

## Abstract

African trypanosomes cause devastating disease in sub-Saharan Africa in humans and livestock. The parasite lives extracellularly within the bloodstream of mammalian hosts and is transmitted by blood-feeding tsetse flies. In the blood, trypanosomes exhibit two developmental forms: the slender form and the stumpy form. The slender form proliferates in the bloodstream, establishes the parasite numbers and avoids host immunity through antigenic variation. The stumpy form, in contrast, is non-proliferative and is adapted for transmission. Here, we overview the features of slender and stumpy form parasites in terms of their cytological and molecular characteristics and discuss how these contribute to their distinct biological functions. Thereafter, we describe the technical developments that have enabled recent discoveries that uncover how the slender to stumpy transition is enacted in molecular terms. Finally, we highlight new understanding of how control of the balance between slender and stumpy form parasites interfaces with other components of the infection dynamic of trypanosomes in their mammalian hosts. This interplay between the host environment and the parasite’s developmental biology may expose new vulnerabilities to therapeutic attack or reveal where drug control may be thwarted by the biological complexity of the parasite’s lifestyle.

## 1. Introduction

**Figure 1:**
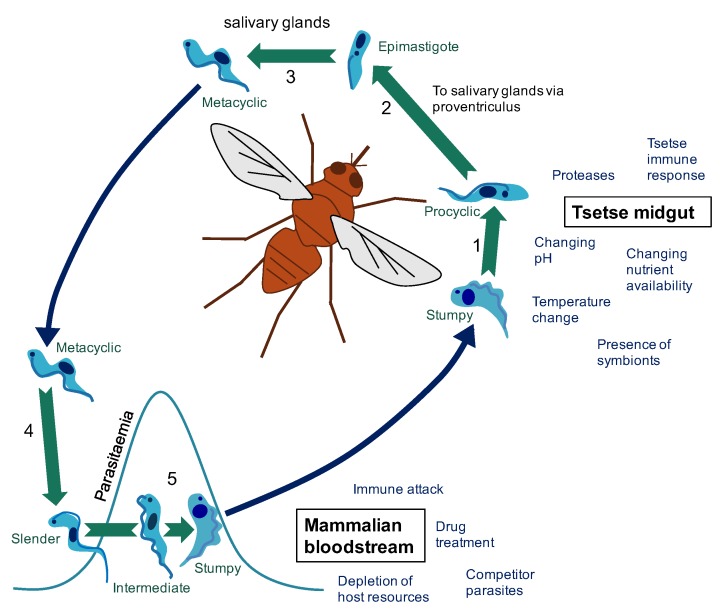
The changing environments of the *T. brucei* life cycle. (1) Following a tsetse fly blood meal on an infected mammal (blue arrow), *T. brucei* stumpy cells reaching the tsetse midgut differentiate to procyclic cells. (2) Development of *T. brucei* proceeds through the tsetse midgut to the proventriculus. (3) Differentiation to epimastigote cells is followed by production of mammalian-infective metacyclic cells in the salivary gland. (4) On transfer to a new mammalian host via a tsetse fly bite (blue arrow), metacyclic cells differentiate to bloodstream forms. (5) In the bloodstream, proliferative slender cells elevate the parasitaemia until accumulation of a density-dependent signal triggers differentiation to the cell-cycle arrested stumpy form (via an intermediate stage). The stumpy form of *T. brucei* faces a particular challenge in that it must survive long enough in the bloodstream to be transmitted, but it must also survive long enough in the tsetse midgut to differentiate to the next life cycle stage and establish infection. These different environments pose different challenges that must be overcome to enable the continuation of the parasite life cycle. For example, in the mammalian host (red section), parasites must evade immune attack to ensure parasite survival and avoid excessive exploitation of host resources to ensure the host survives long enough to enable successful transmission to the tsetse fly vector. On uptake to the tsetse fly midgut (orange section), the parasites face a shift in nutrient availability and temperature among other challenges. For a review of factors effecting establishment of tsetse fly midgut infection see [1].

Persistence in regularly changing environments demands that vector-transmitted pathogens rapidly adapt to fluctuating conditions. *Trypanosoma brucei*, similar to many eukaryotic parasites, encounters different environments as it transitions between its mammalian host and the tsetse fly vector, *Glossina* spp. In these different environments, distinct *T. brucei* life cycle forms, specialised for the various niches encountered, have evolved to optimise parasite survival and to ensure transmission to the next host (Figure 1).

The challenges presented by the distinct environments encountered during the life cycle differ. In the mammalian bloodstream, for example, the parasite must face a host immune response that is constantly trying to control the infection. To evade this control and to thrive extracellularly within the mammalian host bloodstream, trypanosomes have evolved the extraordinary capacity to overcome a barrage of host innate and adaptive immunological defences via the expression, and switching, of their expressed Variant Surface Glycoprotein (VSG) coat, a process termed antigenic variation [2]. The periodic destruction of antigenic variants, followed by the outgrowth of antigenically distinct variants, contributes to the undulating waves of parasitaemia typical of trypanosome infection.

Within each of these cyclical waves, a density-dependent developmental switch represents another important evolutionary adaptation, both regulating parasite virulence and pre-adapting the parasite for uptake by the tsetse fly. Infections are maintained in the host bloodstream by rapidly proliferating morphologically “slender” cells. Allowed to replicate unchecked, slender parasites would rapidly kill the host [3]. Consequently, it is important that parasites regulate their growth in vivo to promote longevity of the host and maximize their transmission potential [4,5]. Such regulation is achieved by a developmental switch initiated upon reaching a critical parasite density. Specifically, proliferative slender cells respond to the accumulation of a small soluble parasite-derived factor, stumpy induction factor (or factors) (SIF), and differentiate to specialized cell-cycle arrested “stumpy” cells [6,7]. As growth-arrested stumpy cells accumulate during the ascending parasitaemia, the parasite population growth is restricted thereby prolonging the infection and increasing the opportunity for transmission [6,7,8]. Parasite strains that respond to this density-mediated growth control by differentiating to stumpy cells are described as pleomorphic. Extensive passage of laboratory strains can, however, select against differentiation to stumpy cells, resulting in “monomorphic” strains that do not arrest as transmissible forms in response to parasite density [9].

Beyond its cell-cycle arrest, the stumpy form of *T. brucei* has characteristics that distinguish it from the slender form, fulfilling its specialised role in transmission to the insect vector. In this review, we begin by describing the developments in our understanding of the nature of the different forms of *T. brucei* in the mammalian host, from initial morphological descriptions to detailed molecular characterisation. Secondly, we discuss investigations into the mechanism coordinating differentiation from slender to stumpy cells, considering how historical findings have provided tools to further elucidate this process. Finally, we reflect on outstanding questions relating to *T. brucei* differentiation and infection dynamics in the mammalian host.

## 2. Characterisation of Slender and Stumpy Cells

Differences in the morphology of *Trypanosoma brucei* spp. in the mammalian bloodstream were recognised in the early 1900s with descriptions and drawings of slender, intermediate and stumpy cells by Bruce [10]. These descriptions were followed by the observation that the stumpy form was apparently the morphotype capable of establishing infection of the tsetse fly vector [11,12]. Subsequent research sought to answer why the stumpy form was able to survive in the tsetse midgut and thus continue the parasite’s life cycle.

On uptake by the vector, parasites enter a challenging environment in the tsetse midgut. Slender cells are killed rapidly and it is stumpy cells that survive to differentiate to the next life cycle stage [13]. Characteristics of stumpy cells that likely contribute to their survival in the fly midgut include their relative resistance to acidic and proteolytic stress [14]. For example, the differential response to mild acid conditions (pH 5.5) has been used as a selection to monitor the differentiation to stumpy cells in some studies [15], as slender cells are sensitive and stumpy cells are resistant. Moreover, protease treatment has been shown to act as a trigger for bloodstream forms to differentiate to procyclic cells [16,17,18]. Likewise, pretreatment in mild-acid conditions (pH 5.5) has been shown to stimulate stumpy cells to differentiate to procyclic cells at 27 °C [19].

Different host environments also provide alternative nutritional opportunities for a parasite. For instance, in the mammalian bloodstream *T. brucei* can exploit an abundant glucose supply. On uptake by the tsetse fly, however, proline replaces glucose as the most readily available energy source [20], and an intact proline metabolic pathway has been shown to be essential for successful *T. brucei* colonisation of the tsetse midgut [21]. Correspondingly, procyclic cells inhabiting the tsetse midgut possess a more elaborate and active mitochondrion than slender bloodstream forms [22]. In preparation for this, differentiation in the bloodstream from the slender form to the stumpy form is accompanied by activation of the mitochondrion, as evidenced by detection of NAD diaphorase activity and the ability of intermediate and stumpy cells to use the Krebs cycle intermediate α-keto-glutaric acid (α-KGA) to maintain motility in the absence of glucose [23]. NADH dehydrogenase (diaphorase) activity has remained an accepted (though qualitative and somewhat ambiguous) marker for identifying stumpy bloodstream cells, but has more recently been superseded by the use of mitochondrial dyes such as MitoTracker^®^ (Molecular probes^TM^) [24]. A further intracellular rearrangement occurring during the differentiation to stumpy cells is the repositioning of the lysosome. In slender cells a single lysosome is positioned equidistant between the nucleus and kinetoplast at the posterior end of the cell, whereas differentiation to stumpy cells is accompanied by movement of the lysosome to the anterior side of the nucleus [25]. An additional remodelling of the endocytic system that occurs during differentiation is the down-regulation of the haptoglobin-haemoglobin receptor in stumpy cells [25].

Once stumpy cells have been ingested by the tsetse fly they must be ready to perceive the signals that trigger differentiation to the proliferative procyclic form adapted for the colonisation of the midgut. Cold shock (i.e., a reduction from 37 °C to 20 °C) is able to sensitise stumpy (but not slender) parasites for perception of the differentiation signals cis-aconitate and citrate [26]. A carboxylate transporter protein family, known as Proteins Associated with Differentiation (PAD), is required for the response to this citrate cis-aconitate differentiation signal [27]. Thus, PAD2 expression is up-regulated at 20 °C and the protein is redistributed from the flagellar pocket to the parasite surface, enabling the cold-stimulated perception of the differentiation signal. PAD1 protein, in contrast, was found to be expressed specifically on the surface of stumpy, but not slender, bloodstream cells and is now used extensively in differentiation studies as a molecular marker for stumpy parasites. For example, in a study of chronic *T. brucei* infections in mice, intermediate and stumpy cells were found to dominate later stages of infection [8], the relative expression of PAD1 mRNA detected by qRT-PCR being used as a tool to reflect the transmission capacity of the population. PAD1 mRNA expression is detectable in the intermediate cells that precede morphological transformation to stumpy cells, this being followed by protein expression as mature stumpy cells arise, the regulatory signals governing progressive activation having been localised in the PAD1 3′UTR [28].

Another group of proteins with strong developmental regulation are the Expression Site Associated Gene (ESAG) 9 family [29] which are specifically expressed in stumpy, but not slender cells. Although originally identified as components of some VSG expression sites [30], ESAG9 genes are rare expression site (ES) components [31], being frequently found in non-expression site locations. The function fulfilled by this family of proteins remains unclear, but they appear to be secreted by the parasite and could therefore interact with the mammalian immune system or assist stumpy form transmission upon entry into the fly. Regions in the 3′UTRs of ESAG9 [32] and PAD1 [28] with involvement in the developmental regulation of these stumpy-expressed proteins have been identified. Moreover, a cell line engineered to place expression of a reporter gene under the control of the PAD1 3′UTR has been developed as a useful tool to study differentiation from slender to stumpy cells. This cell line was successfully used in a high-throughput screen to identify a compound able to induce partial differentiation to stumpy cells [33], whereas a PAD1 3′UTR linked GFP reporter has also been used in cytological studies of stumpy cells in adipose tissue [34]. Recently, knowledge of the stumpy-specific regulation of ESAG9 was successfully applied to identify a protein that negatively regulates the expression of stumpy-specific genes [35]. This utilised a screen exploiting a cell line containing both a tetracycline-inducible genome-wide RNAi library [36] and a drug resistance gene under the control of the 3′UTR of ESAG9. The screen identified REG9.1, a molecule with a predicted RNA binding motif, whose silencing by RNAi resulted in derepression of many ESAG9 genes as well as the mRNAs for two novel predicted cell-surface families (Family 5 and Family 7) that were also found to be enriched in stumpy cells [35].

The most obvious distinction between slender and stumpy cells of *T. brucei* is that a slender population is proliferative, whereas stumpy cells are arrested in G0/G1 of their cell cycle [37]. In addition to extending host survival, the cell cycle arrest displayed by stumpy cells contributes to their competence for transmission. For instance, it was proposed that there was a point in the cell cycle (G0/G1) when cells were receptive to the signal to differentiate to procyclic cells after which they re-enter the cell cycle [38]. This co-ordination of cell cycle position and differentiation capacity ensures that the organellar repositioning events that accompany differentiation can be achieved without perturbation of the precisely orchestrated cytological events that accompany the parasite’s cell division [39,40]. It also explains why uniform populations of stumpy cells already arrested at this point of the cell cycle are able to differentiate synchronously, unlike asynchronous monomorphic bloodstream forms [41].

The efficiency with which stumpy cells differentiate synchronously to procyclic cells in vitro provides a useful and physiologically relevant assay to monitor the abundance of stumpy cells in a bloodstream parasite population. This assay can be effectively performed in vitro using cis-aconitate, combined with a reduction in temperature from 37 °C to 27 °C, to stimulate differentiation [41,42,43]. The changes accompanying differentiation to procyclic cells include replacement of the VSG coat with a procyclin surface coat [44,45,46], repositioning of the mitochondrial genome (the kinetoplast) [40] and cell cycle re-entry [38,47]. Thus, the tools exist to effectively test the capacity of a population of bloodstream trypanosomes to synchronously differentiate to procyclic cells, helping to distinguish physiologically relevant stumpy cells from (for example) RNAi phenotypes that perturb cell shape or proliferation and generate cells superficially similar to stumpy morphology.

Differentiation from stumpy to procyclic cells triggered by citrate/cis-aconitate or mild acid treatment is signalled via a protein phosphorylation cascade [18]. *Tb*PTP1, a protein tyrosine phosphatase [48], dephosphorylates and inactivates the DxDxT class serine-threonine phosphatase *Tb*PIP39 (*Tb*PTP1-interacting protein, 39kDa) [49] to prevent differentiation of stumpy cells in the bloodstream. Once parasites reach the appropriate environment of the tsetse midgut, they perceive the differentiation signal, *Tb*PTP1 is inactivated and *Tb*PIP39 is phosphorylated (and so activated) and translocated to the glycosome. *Tb*PIP39 mRNA is expressed at highest levels in stumpy cells. However, while barely detectable in slender cells, *Tb*PIP39 protein is detected in stumpy cells but protein expression is highest in procyclic cells [49]. The expression of proteins, such as *Tb*PIP39, required for differentiation to the next life cycle stage is another feature of stumpy cells that prepares them for transmission. Indeed, the detection of increased *Tb*PIP39 transcript abundance has been used as an indicator of the presence of stumpy cells in a bloodstream population of *T. b. rhodesiense* [50], and this can supplement the use of other stumpy markers such as PAD1 expression. Likewise, the protein kinase NRKA/B is a key regulator of differentiation when parasites enter the tsetse fly and this molecule is exclusively expressed in stumpy cells, providing another means to identify this cell type [51,52].

Further differences between slender and stumpy bloodstream cells have been described in how these cells interact with the host immune system. Slender cells of *T. brucei* can switch their expressed VSG to one that is antigenically distinct and outgrow as new antigenic variants. This enables survival of a subset of switched cells when the host develops an immune response against the dominant VSG expressed by the population ([22], and reviewed in [53]). Stumpy cells, in contrast, do not switch to express novel VSG [54] and—being irreversibly arrested—cannot establish new variant populations. However, stumpy cells have a higher rate of endocytosis than slender cells and are better able to rapidly clear IgG-VSG from the cell surface to delay complement-mediated lysis [55,56]. This may enable stumpy cells to survive for long enough to increase their probability of transmission.

*T. brucei* VSG expression is under strict control with transcription active from only one expression site at a time, maintaining monoallelic VSG expression [31,57]. A link between antigenic variation and life cycle progression was proposed when it was found that overexpression of an exogenous VSG in monomorphic *T. brucei* could stimulate an accumulation of the cell population in G1 of the cell cycle, expression of PAD1 and an enhanced ability to differentiate to procyclic cells in response to cis-aconitate treatment [58]. This effect was mediated through repression of the active ES, and linked to the concomitant silencing of ESAGs 1, 2 and 8 since RNAi targeting these transcripts reproduced the phenotype. However, this effect must be distinct from the quorum-sensing mediated differentiation from slender to stumpy cells since the phenotype caused by overexpression of the exogenous VSG was reversible. This contrasts with density-induced slender to stumpy differentiation which is irreversible.

Encouraged by the findings in monomorphic cells, Zimmermann et al. (2017) recently tested the hypothesis that transcriptional attenuation at the ES could elicit life cycle progression in biologically-relevant pleomorphic cells in vitro [59]. Here, ectopic overexpression of a second VSG generated two populations of cells with distinct growth phenotypes, either those which rapidly ceased proliferation upon induction or those that continued to divide. The growth-arrested cells exhibited complete transcriptional attenuation of the ES, whilst those that continued to divide did not. Moreover, VSG overexpression-induced ES attenuation caused cells to accumulate in G1 and upregulate the expression of stumpy-specific proteins, such as PAD1, in a density (and therefore SIF) independent manner. Upon exposure to cis-aconitate, cells could synchronously differentiate to procyclic cells, and were capable of establishing mature tsetse fly infections demonstrating their physiological relevance. Cells which remained proliferative upon overexpression of a second VSG, however, remained slender and, though the initial VSG was silenced, the ES remained in an incompletely attenuated state. The authors proposed that, upon switching to a new ES with defective function, the parasite may respond in one of two ways: if the activity of the initial ES remains above a critical threshold, this state is maintained and the parasite continues to proliferate; if, however, the ES-activity has fallen below this threshold, the parasite differentiates to the growth-arrested stumpy form in a density-independent manner. This mechanism was proposed to provide a potential escape for parasites that switch to an ES insufficient to fulfil requirements in a given host environment, although the physiological relevance of this for overall parasite transmission remains to be established.

Advances in technology have allowed global analyses of differences between life cycle forms in terms of expressed transcripts, protein abundance and phosphorylation status. In the stumpy form, reduced protein synthesis, possibly regulated through translation initiation [60], accompanies cell cycle arrest [61]. Accordingly, transcripts such as histones associated with an active cell cycle, as well as glycolytic pathway components and cytoskeletal transcripts were found to be down-regulated in stumpy cells [62,63,64]. On the other hand, transcripts involved in protein synthesis were observed to be up-regulated in procyclic cells [62]. Transcripts escaping the generalised translational repression in stumpy cells were identified from polysomal material [61]. Thus, components of oxidative phosphorylation and amino acid metabolism, amino acid transporters and mitochondrial transcripts were enriched in stumpy cells, matching expectation for the life cycle stage primed for transmission to the insect vector.

In *T. brucei* transcript levels do not necessarily correspond to protein levels, as much regulation of gene expression occurs post-transcriptionally through binding of regulators to (predominantly) the 3′UTR of a particular gene, with consequences for transcript stability and translational efficiency [65]. Proteomic analyses are therefore required to compare the protein composition of various *T. brucei* life cycle forms. RNA-binding proteins, cell surface proteins and proteins involved in mitochondrial and glycosomal energy metabolism have been found to vary in abundance between cultured bloodstream and procyclic form *T. brucei* [66,67]. Such studies often used SILAC (stable isotope labelling by amino acids in culture) and this methodology has been extended to analysis of protein abundance in slender, stumpy and procyclic cells of the pleomorphic cell line *T. brucei* AnTat 1.1 [68]. Here, it was observed that mitochondrial proteins were more abundant in stumpy and procyclic cells than in slender cells, with many protein abundance changes already being evident between the slender and stumpy stages. It is important to note that the increase in abundance of mitochondrial proteins in procyclic cells observed in this study was of a greater magnitude than in a previous study [67] that did not use a pleomorphic strain of *T. brucei*. This emphasises the need to use pleomorphic strains, competent to complete the life cycle, to fully characterise the features of different life cycle forms. Further insight into the timings of protein abundance changes during the *T. brucei* life cycle has been obtained by following changes in protein abundance and phosphorylation before and after the point of commitment to differentiation from bloodstream stumpy cells to procyclic cells [51]. Just 3 h following stimulation of differentiation with cis-aconitate, proteins associated with the procyclic form cell surface and developmental changes in metabolism were upregulated. Additionally, a change in the phosphorylation status of eukaryotic initiation factors was detected consistent with reinitiation of protein synthesis early in differentiation [62]. Likewise, also using the synchronous differentiation of stumpy cells, Dejung et al. found proteins indicative of a changing cell surface and metabolism increased in abundance 2 h following induction of differentiation [69]. Moreover, some proteins enriched in procyclic cells, such as PAD2 and *Tb*PIP39 were already more abundant in stumpy cells relative to slender cells.

**Figure 2:**
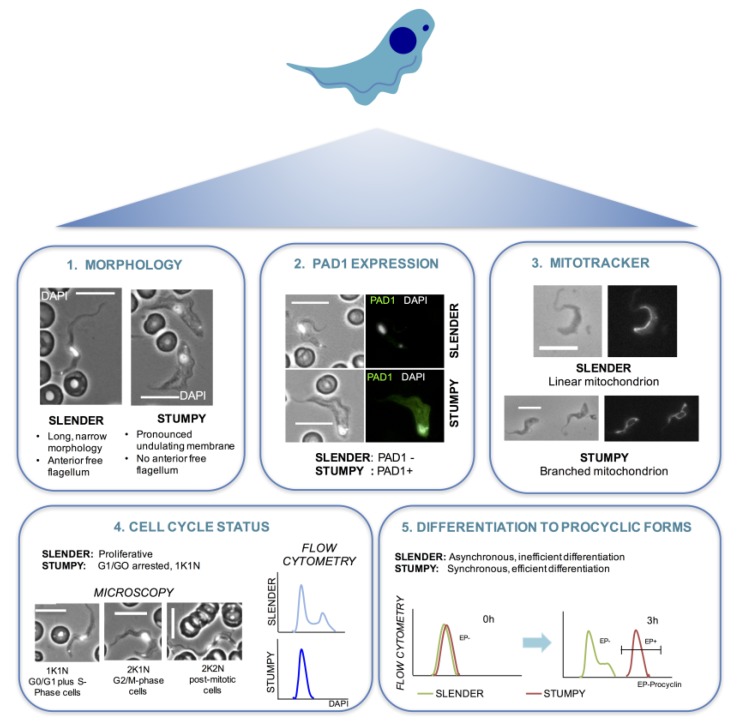
Characteristics of a stumpy cell. A number of assays can be used to test the proportion of cells in a *T. brucei* bloodstream form population that are slender or stumpy. (1) Firstly, stumpy cells are clearly morphologically distinct from slender cells. The representative cells shown have been labelled with the DNA stain DAPI (white) to highlight the position of the nucleus and kinetoplast in each case. (2) Secondly, stumpy cells express the PAD1 protein on their surface [27]. PAD1 positive cells can be identified using the PAD1 antibody by immunofluorescence microscopy or Western blotting. PAD-based reporter assays have also been established for enzymatic or cytological quantitation. (3) Stumpy cells also have a more elaborated mitochondrion than slender cells [23] and this can be detected by incubating the cells with mitotracker dye [24] before fixing and visualising the cells with a fluorescence microscope. (4) During the cell cycle, *T. brucei* cells first segregate their replicated mitochondrial genome (kinetoplast, K) and then their nucleus (N) prior to cytokinesis to generate two daughter cells [71]. Stumpy cells are arrested in G1/G0 of the cell cycle and have 1 kinetoplast and 1 nucleus (1K1N) [37]. Thus, differentiation to a stumpy population is accompanied by an accumulation of 1K1N cells, and this can be detected by staining with an appropriate DNA dye (e.g., DAPI) and KN scoring by immunofluorescence microscopy or flow cytometry (a completely stumpy population will have a single dominant peak corresponding to G1/G0). (5) Finally, stumpy cells, unlike slender cells, are able to differentiate synchronously to procyclic cells in culture [41], when differentiation is triggered by incubating cells at 27 °C in SDM-79 medium containing 6 mM cis-aconitate. Differentiation to procyclic cells is indicated by expression of EP procyclin [72] and can be detected using an EP procyclin antibody and flow cytometry. Maximal expression of EP procyclin is detectable 3 h after exposure of stumpy cells to cis-aconitate [51].

As a consequence of the extensive differences in the characteristics and developmental potential of slender and stumpy cells of *Trypanosoma brucei* it is important to be able to identify these forms within a population of trypanosomes under study (Figure 2). For instance, in cases where variations in gene expression over time or in different conditions are being reported in bloodstream parasites it is essential to consider whether perceived differences may be a result of varying proportions of differentiated cells, which may or may not be a consequence of the conditions under test. For example, in the recent description of transcripts in *T. brucei* that show circadian control [70], a reporter cell line with GFP expression under the control of the PAD1 3′UTR was used to confirm that the majority of the trypanosome cultures under study were slender (GFP-negative). This confirmed that variations in the transcript abundances observed were not contributed by variations in the proportion of stumpy cells.

## 3. Tools to Understand Life Cycle Development

In parallel to the extensive characterisation of slender and stumpy form parasites, there has also been significant progress in understanding of the mechanisms regulating differentiation from slender to stumpy cells. This has been assisted by tools and technologies that enable dissection of the underlying processes.

Early differentiation work focused on the anti-proliferative properties of the repurposed anti-cancer drug DL-α-difluoromethylornithine (DFMO), a specific irreversible inhibitor of the enzyme responsible for catalysing the first reaction in the polyamine biosynthesis pathway, ornithine decarboxylase (ODC) [73,74]. Exposure of mammalian cells to DFMO causes a rapid depletion of intracellular levels of the polyamines spermidine and putrescine resulting in the cessation of cellular proliferation, though largely in the absence of cell death [73,75]. Furthermore, many studies have attested to the importance of the role that polyamines, in particular spermidine and putrescine, play in the stimulation and regulation of mammalian cell differentiation (reviewed in [76]). Based on these observations, early studies sought to explore the possibility that polyamine depletion upon DFMO treatment could function to regulate *T. brucei* slender to stumpy differentiation, and, moreover whether DFMO induced differentiation events, and the subsequent morphological and physiological changes, were relevant to those seen in natural pleomorphic populations [77]. After treatment with DFMO, monomorphic *T. brucei* strains, grown either in vitro or in vivo, quickly became deficient in polyamines and stopped proliferating with no apparent cytotoxic effect [77,78,79,80]. In addition, monomorphic slender cells transformed morphologically to a smaller rounder cell form with a shortened flagellum, suggestive of a stumpy-like form [77]. While Bacchi et al. [78] were unable to isolate true stumpy cells from DFMO treated animals, possibly due to clearance by the immune system, de Gee et al. [79] demonstrated enhanced activity of NAD diaphorase in monomorphic cells isolated from infections following DFMO treatment, suggesting activation of the mitochondrion in preparation for transmission to the tsetse fly. Giffin et al. [77] reported similar morphological and physiological changes in response to DFMO when culturing monomorphic strains in vitro. However, though the DFMO-treated cells appeared to have increased mitochondrial activity and some stumpy characteristics, they were not able to efficiently transform to procyclic cells in SDM79 medium [77,80]. Moreover, treatment with an exogenous source of putrescine was shown to relieve the effects of DFMO treatment allowing cells to successfully re-enter proliferation [77] contrasting with the irreversible and uniform cellular quiescence in G0/G1 [37] of stumpy cells. The DFMO response therefore does not fully resemble physiological differentiation.

A similar non-physiological response which superficially resembled the natural slender to stumpy differentiation process was observed by Penketh et al. [81] following repeated exposure of cells to low concentrations of the methylating agent 1,2-bis(methylsulfonyl)-1-methylhydrazine. Monomorphic cells treated either in vitro or in vivo morphologically resembled stumpy cells within 48 h, expressed elevated mitochondrial activity and were able to establish stable procyclic cultures when transferred to Cunningham’s Medium. Despite the eventual accumulation of G0/G1 arrested cells upon exposure, analogous to events in natural populations, this was not before progression through a transitional stage that appeared defective in cytokinesis. Specifically, large numbers of multinucleated cells (3–4 nuclei/cell) predominated before semi-synchronous progression to a G0/G1 arrested population [6,81] indicating 1,2-bis(methylsulfonyl)-1-methlhydrazine was not useful as a tool for physiologically-relevant differentiation studies. Cell cycle arrest and morphological transformation to stumpy-like cells has also been observed in vivo following treatment with a small molecule inhibitor of Clan CA cysteine proteinases, carbobenzoxy-phenylalanyl-alanine-diazomethyl ketone (Z-Phe-Ala-CHN_2_) [82]. Though pleomorphic cells arrested in their growth (without an accumulation of multinucleated cells) and displayed stumpy-like morphology following treatment with the inhibitor, this was accompanied by a significant enlargement of both the lysosome and the parasite, and decreased protein degradation, suggesting that the Z-Phe-Ala-CHN_2_ response is also non-physiological [82].

An important breakthrough came in 1997 when Vassella et al. described a novel experimental tool which provided insight into the mechanism of stumpy differentiation [6]. Prior to this study, it had been speculated that the slender to stumpy differentiation was triggered by the accumulation of an exogenous growth inhibitor upon reaching a critical cell density, as demonstrated by the ability of plasma from infected animals to inhibit cell proliferation in vitro [83] and the observation that pleomorphic cells would arrest and differentiate to stumpy cells above particular cell densities [6,84]. An elegant series of experiments utilising conditioned medium prepared from bloodstream form cultures at high density demonstrated that parasite density alone induced differentiation, thus excluding cellular programming, depletion of essential nutrients or cell-to-cell contact as possible initiators of the slender to stumpy transition. Pleomorphic cells cultured in the presence of conditioned medium rapidly and uniformly arrested in G0/G1 (without accumulating aberrant cell cycle configurations), morphologically became stumpy, and exhibited NADH diaphorase activity. The effect of the conditioned medium was attributed to a soluble, heat stable, ≤500 Da molecule termed SIF, the identity of which remains elusive [6]. Significantly, SIF-induced differentiated cells were capable of synchronous differentiation to procyclic cells upon a temperature drop and exposure to cis-aconitate, further demonstrating the physiological relevance of the differentiation events induced by exposure to conditioned medium. Interestingly, laboratory-adapted monomorphic cells, incapable of natural differentiation to stumpy cells in vivo, were less responsive to SIF; however conditioned medium prepared from these cell lines was equally as effective as that from pleomorphic cells at inducing cell cycle arrest and differentiation to stumpy cells in differentiation-competent recipients. As such, this suggested that monomorphic cells produce functional SIF, but are in some form defective in either receiving or transducing the SIF signal through a competent pathway [6].

The SIF response pathway represented the next piece in the puzzle. Intracellular levels of cyclic 3′, 5′-adenosine monophosphate (cAMP) had provoked interest following the demonstration of its presence and fluctuation during the course of in vivo slender to stumpy differentiation [85,86]. During infection, the intracellular cAMP content of parasites was shown to increase over three-fold as the parasitaemia reached peak density, then returned to basal levels as stumpy cells accumulated [87]. Hypothesizing a model whereby the density-dependent production of SIF triggers the cAMP signalling pathway to induce differentiation, Vassella et al. [6] exposed pleomorphic cultured cells to the membrane-permeable cAMP analogue 8-(4-chlorophenylthio)-cAMP (8-pCPTcAMP). This generated rapid cell cycle arrest, induction of stumpy morphology, activation of the mitochondrion and accumulation of cells in G0/G1 with near identical kinetics to that of SIF-induced differentiation, with no cytotoxicity. Moreover, intracellular cAMP levels were shown to increase 2–3-fold upon incubation of cells with SIF [6]. Thus, the cAMP signalling pathway was considered a conceivable inducer of slender to stumpy differentiation which responded to the density-mediated accumulation of SIF. The capacity for 8-pCPTcAMP to induce differentiation events in monomorphic cell lines [88] provided further support for the differentiation promoting properties of cAMP, and led to the hypothesis that 8-pCPTcAMP could be a useful tool to identify the genes involved in slender to stumpy differentiation.

Whilst there was little doubt that 8-pCPTcAMP could trigger differentiation events analogous to those initiated by SIF, the perception that cAMP played an important role in the induction and regulation of slender to stumpy differentiation remained in question, as no direct cAMP-dependent effector proteins were annotated in the genome [89]. Ultimately, Laxman et al. [89] were able to demonstrate that the events observed by Vassella et al. [6] were attributable to the potent activity of the hydrolysis product of 8-pCPTcAMP (8-pCPT-2′-O-Me-5′-AMP) rather than 8-pCPTcAMP itself. This was achieved by comparing the effects of membrane-permeable and hydrolysis-resistant 8-pCPTcAMP analogues on cell proliferation [89]. Significantly, this demonstrated that cAMP itself was not involved in the induction of the slender to stumpy differentiation pathway, but rather a hydrolysis product of it, such as AMP.

For differentiation or infection studies to be physiologically relevant, pleomorphic cell culture and genetic manipulation methods required development and optimization. The first report of successful continuous in vitro culture of a pleomorphic cell line came in 1979 when Brun et al. used a mammalian feeder cell layer consisting of a rabbit fibroblast-like cell line grown in modified MEM and supplemented with rabbit serum [90]. Today, the standard method of pleomorphic cell culture involves maintenance of cells densities < 1 × 10^6^ cells/mL [91] for minimal periods of time in HMI9, a medium based on Iscoves modified DMEM medium supplemented with bathocuproinedisulphonic acid, L-cysteine, hypoxanthine, 2-mercaptoethanol, pyruvate, thymidine and foetal bovine serum [92]. For many pleomorphic cell lines the provision of methyl cellulose also assists prolonged culture [84]. Building on the progress in establishing protocols for efficient procyclic [93] and monomorphic [94,95] transfection, MacGregor et al. demonstrated the usefulness of Amaxa Nucleofector technology when applied to pleomorphic cell transfection [91]. Shown to increase the transfection efficiency of monomorphic cells 1000-fold [95], this technology increased the transfection efficiency of pleomorphic AnTat 1.1 90:13 cells [26] to 10^−7^–10^−6^ thus permitting routine genetic manipulation of cells capable of a full life cycle progression.

With advances in the repertoire of genetic experimental tools available to study *T. brucei*, for example the discovery of an active RNAi pathway [96], improved transfection efficiencies [95,97] and the publication of the Tritryp *T. brucei* genome in 2005 [98], it became possible to perform high throughput gene function analyses. In particular, Alsford et al. described a novel, high resolution experimental approach termed RNA Interference Target Sequencing (RITseq) which would allow for genome-wide analysis of fitness upon induction of RNAi-mediated gene knockdown [36]. An inducible RNAi library, providing around five-fold *T. brucei* genome coverage [99] was targeted to a unique tagged rRNA locus [97] with an inducible meganuclease cleavage site [36,94]. Cell growth under RNAi-induction led to a loss of representation of target sequences within the remaining proliferating population when RNAi-mediated gene knockdown conferred a fitness deficit. Reads from Illumina sequencing of PCR-amplified RNAi cassette insert DNA could be mapped back to the genome, identifying “cold spots” representative of areas of the genome which were essential for growth in the surveyed assay conditions [36]. Since its description, RITseq has proven to be a phenomenally effective tool in both functional analyses and drug discovery settings. Essential genes for various life cycle stages and events, mechanisms of antitrypanosomal drug action and resistance, and mitochondrial proteins essential for normal cell growth and division have all been identified using RITseq [36,100,101].

## 4. Molecular Components of the Slender to Stumpy Regulatory Pathway

By combining RITseq technology and selection using differentiation-inducing 8-pCPTcAMP/AMP, Mony et al. were able to identify and validate many intracellular components of the signalling pathway driving stumpy formation [102]. Monomorphic *T. brucei* populations, transfected with the RNAi library, were exposed to membrane permeable 8-pCPTcAMP/AMP and grown in vitro with or without RNAi induction. Uninduced populations became non-proliferative and underwent cell death in response to 8-pCPTcAMP/AMP. In contrast, the induced population parasites, where components of the signalling pathway had been silenced by RNAi, continued to proliferate in the presence of the differentiation signals. Thereafter, RNAi insert DNA was amplified from the 8-pCPTcAMP/AMP resistant, differentiation defective populations and subjected to Ion Torrent deep sequencing, with reads aligned to the trypanosome genome. The screen identified an extensive cascade of molecules, together assembling a complex signalling pathway driving slender to stumpy differentiation. Groups of identified genes formed distinct tiers of a signalling pathway, from protein kinase and phosphatase signal transducers (e.g., *YAK* (*Tb*927.10.15020) and *PP1* (*Tb*927.4.3620/30/40)) to RNA-binding effector molecules such as *RBP7* (*Tb*927.10.12100) [102,103]. Critically, whilst the RNAi library cell line used in the screen to identify pathway components was monomorphic, the physiological relevance of the genes identified by Mony et al. [102] was verified in pleomorphic cell lines. Thus, nine of the genes were tested for sensitivity to SIF upon RNAi induction in vivo. In contrast to the AnTat 1.1 90:13 parental cell line which underwent cell cycle arrest and differentiation to transmission competent stumpy cells, all but two of the induced RNAi lines generated in the study (*ADSL* (*Tb*927.9.7550) and *ADSS* (*Tb*927.11.3650)) showed increased resistance to SIF, revealed by a delayed or defective differentiation phenotype. Thus, induced cell populations retained slender morphology, showed reduced expression of the stumpy marker PAD1, did not enrich in G0/G1 arrested cells and lacked an elaborated mitochondrion. Mony et al. [102] were therefore able to utilize a potent combination of classic and newly developed technologies and tools to provide information on the genes required to differentiate from proliferative long slender bloodstream forms to transmission competent quiescent stumpy cells [103].

In addition to the aforementioned signal transducers *YAK* (*Tb*927.10.15020) and *PP1* (*Tb*927.4.3620/30/40), the α2 subunit of *AMPK* (*Tb*927.3.4560) was identified by Mony et al. [102] in the genome-wide RNAi screen. *AMPK*, a fundamental regulator of energy homeostasis, is activated in response to alterations in the cellular AMP/ATP ratio. In mammalian cells grown in stress conditions, a drop in ATP levels, and a corresponding rise in cellular AMP, triggers AMPK to phosphorylate downstream targets, thus coordinating ATP generating and consuming processes to bring about energy balance [103,104]. Mammalian *AMPK* is an inhibitor of the evolutionarily conserved protein kinase target of rapamycin (mTOR), a regulator of cell growth, cell cycle, stress response and energy balance [105,106]. Characterisation of the trypanosome TOR like proteins, *Tb*TOR1 and *Tb*TOR2, revealed central functions, analogous to those of their mammalian counterparts, in protein synthesis and cell growth (*Tb*TOR1) and in maintaining cytokinesis (*Tb*TOR2) [107]. Interestingly, however, the structurally and functionally distinct *Tb*TOR4 paralog was described to function as part of a larger complex with *Tb*LST8, MVP and *TbArmtor* (*Tb*TORC4) to negatively regulate slender to stumpy differentiation [106], thus forming a potential link to the identified *AMPK* in the Mony et al. screen [102]. Upon induction of *TbTOR4* RNAi in monomorphic cultures, cells exhibited hallmarks of slender to stumpy differentiation: morphological change; an irreversible accumulation of cells in G0/G1; mitochondrial elaboration; and the ability to establish procyclic cultures upon treatment with cis-aconitate. Upon *Tb*TOR4 knockdown, mRNA and protein analysis showed a subtle increase in PAD1 expression, though not to the same extent as wild type pleomorphic cells undergoing differentiation. Treatment with 8-pCPTAMP also led to a rapid decrease in *Tb*TOR4 protein levels, supporting the argument against a cAMP dependent pathway and establishing a model where high levels of cellular AMP could activate AMPK which in turn would inhibit the negative regulator *Tb*TOR4, thus allowing the developmental switch from slender to stumpy cells to occur [106]. In addition to *Tb*TOR4, further negative regulators of slender to stumpy form differentiation have been identified, such as MAPK5 [108] and a zinc-finger kinase, ZFK [109]. Though a ZFK null mutant demonstrated reduced growth and increased expression of stumpy markers in vitro the phenotype was not observed in vivo [109]. A MAPK5 null mutant, however, prematurely differentiated to stumpy cells in vivo [108].

More recently, Saldivia et al. [15] have suggested that AMPKα1, and not the AMPKα2 that was identified in the Mony et al. screen [102], is associated with the positive regulation of stumpy form development. In vitro phosphorylation analysis using an α-phospho-Thr172 antibody showed that AMPKα1 phosphorylation was significantly upregulated upon treatment with 8-pCPTAMP, whereas AMPKα2 phosphorylation levels were unaffected. In addition, upon AMPKα1 phosphorylation, levels of stumpy specific transcripts, such as PAD1 and PAD2, were increased and *Tb*TOR4 protein levels were decreased. Moreover, during in vivo analysis with a pleomorphic strain, AMPKα1 was not found be phosphorylated in slender form parasites, whereas its phosphorylation was detected early during differentiation and this was maintained until the population had fully differentiated to stumpy cells. A role for AMPKα1 was further supported by the observation that its phosphorylation was abrogated upon treatment of mice with the AMPK inhibitor compound C, although the specificity of the inhibitor activity was not proven.

## 5. Future Considerations

With improved molecular descriptions of *T. brucei* stumpy cells and tools to disrupt or stimulate the differentiation step from slender to stumpy cells, we are now well placed to investigate the signals contributing to *T. brucei* differentiation in the mammalian host. The availability of cell lines able to report on stumpy formation through PAD1 3′UTR control [28], for example encoding a GFP reporter with a nuclear localisation signal and PAD1 3′UTR [34,58,59], or the use of a PAD1 antibody [27] enables the quantification of stumpy cells in a population. Furthermore, knowledge of the signalling components regulating differentiation [102] has made possible the generation of signal-blind cell lines which do not respond to the quorum-sensing signal. Together such tools could help to identify signals triggering slender to stumpy differentiation through the quorum-sensing signalling pathway, perhaps guiding the identification of the elusive SIF. Regardless of this, the progress made in understanding the molecular control of differentiation in the mammalian bloodstream has revealed new research opportunities and therapeutic implications, as detailed below.

Firstly, understanding of the mechanisms coordinating slender to stumpy differentiation could be employed to force parasites to differentiate prematurely allowing clearance by the host. For example, a differentiation-mediated therapeutic option has already been considered, with compounds capable of inducing slender to stumpy [33] or slender to procyclic form differentiation [110] having been identified in screens using either PAD1 or procyclin 3′UTR regulated reporter cell lines. With identification of the core molecular regulators of differentiation, more targeted therapeutic approaches may now be explored.

Secondly, given the differential sensitivity of slender and stumpy bloodstream parasites to immune attack [55], mild-acid and proteases [14], it is possible that the different biology of stumpy cells makes them more resistant to trypanocidal drugs than slender cells. In the case of *Plasmodium falciparum*, resistance to the antimalarial drug artemisinin was attributed to persister populations generated by the cell cycle arrest of ring stage parasites, with such parasites able to resume development following removal of drug pressure [111]. The difference in the case of *T. brucei* is that stumpy cells are irreversibly arrested in their cell cycle and cannot resume development in the bloodstream. Therefore, a drug treatment that killed slender cells but left surviving stumpy cells would not result in recrudescence of the parasites although transmission might still be possible in the short term. Nevertheless, the metabolism or drug sensitivity of monomorphic cells may not fully reflect the sensitivity or resistance of pleomorphic parasites and new therapeutics should be screened on field-relevant strains rather than laboratory adapted lines.

Thirdly, it will be necessary to understand the development from slender to stumpy cells in the context of different mammalian hosts. For example, although stumpy cells were observed to be dominant in *T. brucei* chronic infection of mice [8], the differentiation status of *T. brucei* during chronic cattle infection, where parasitaemias are often lower, has not been carefully quantitated. Hence, fly infectivity in acute and chronic stages of infections [112] should be correlated with the proportion and abundance of slender and stumpy cells to determine the relevance of development for the epidemiology of animal African trypanosomiasis (AAT).

Finally, *Trypanosoma brucei* bloodstream forms can invade beyond the bloodstream and into tissues. In late stages of infection parasites can be found within the brain parenchyma [113]. Recently, other reservoirs of *T. brucei* have been described including the adipose tissue [34] and the skin [114,115]. Adipose tissue forms of *T. brucei* were shown to have up-regulated expression of genes involved in fatty acid metabolism, indicating an adaptation to that tissue environment. Additionally, trypanosomes inoculated into skin by tsetse fly bite were found to proliferate within the dermis with close interactions between adipocytes and trypanosomes observed by scanning electron microscopy [115]. In both cases, stumpy cells were identified in addition to those in the circulating bloodstream. Hence, trypanosomes resident in the skin or adipose tissue may contribute to transmission in cases where parasitaemia is very low, as can be the case in chronic infections of sylvatic reservoirs. A further consideration is that the nature of different tissue compartments may affect the diffusion and accumulation of a quorum-sensing signal involved in density-dependent differentiation. Thus, it remains to be determined whether transmissible cells have migrated into the tissue following differentiation in the bloodstream or have undergone differentiation within the tissue. Likewise, the extent of flux between compartments throughout the duration of the infection is currently unknown.

## 6. Conclusions

The development of *T. brucei* in the mammalian host is likely coordinated through a complex network of interactions with quorum-sensing mediated density control modulated by different host environments whether this be different host species or different compartments within one host. Moreover, in field settings, the chronic nature of African trypanosome infections often results in co-infections between *T. brucei* and the other African trypanosome species, *Trypanosoma congolense* and *Trypanosoma vivax* [116,117,118]. Thus, the presence of a competitor species of African trypanosome could also contribute to the *T. brucei* differentiation dynamic in the field. We now have a more detailed picture of the biology and molecular regulation of differentiation in the host bloodstream. Future research will need to build on these discoveries and consider how they apply to the economically important livestock infections and how they might impact on the zoonotic potential of human infective parasites in reservoir hosts. The detailed dissection of the signals mediating trypanosome differentiation within the mammalian host may be challenging, but will provide fascinating insight into the intricate biology tying these parasites to their host, and potentially cohabiting species. This in turn may suggest innovative interventions capable of combatting these persistent pathogens.
